# INcreasing Adolescent social and Community supporT (INACT): Pilot study protocol

**DOI:** 10.1371/journal.pone.0317823

**Published:** 2025-03-26

**Authors:** Daniel Hayes, Alexandra Burton, Feifei Bu, Neil Humphrey, Pamela Qualter, Emeline Han, Lou Sticpewich, Joely Wright, Jessica K. Bone, Sophia Maguire, Lucas Caetano Gonzalez Umpierrez, Emily Stapley, Marc S. Tibber, Daisy Fancourt

**Affiliations:** 1 Research Department of Behavioural Science and Health, Institute of Epidemiology and Health Care, Social Biobehavioural Research Group, University College London, London, United Kingdom; 2 Manchester Institute of Education, School of Environment, Education and Development, The University of Manchester, Manchester, United Kingdom; 3 Evidence Based Practice Unit, University College London and the Anna Freud Centre, London, United Kingdom; 4 Department of Clinical, Educational and Health Psychology, Division of Psychology and Language Sciences, London, United Kingdom; Public Library of Science, UNITED KINGDOM OF GREAT BRITAIN AND NORTHERN IRELAND

## Abstract

**Background:**

Social prescribing is a mechanism for connecting patients with non-medical forms of support within the community and has been shown to improve loneliness. Yet uptake from young people (YP) has been lower than for adults. That is thought to be the case because young people are less likely to engage with primary care for wellbeing support, where social prescribing is based. The INACT study will pilot a social prescribing pathway via schools to support young people who are lonely, testing its feasibility and acceptability of delivering, and evaluating its impact on loneliness through a randomised controlled trial.

**Methods:**

This pilot study utilises a two-group (intervention vs. active control) parallel randomised design, with YP as the unit of randomisation. Approximately 78 pupils reporting loneliness will be recruited across 12 mainstream (6 primary and 6 secondary) schools in England. The co-produced social prescribing intervention includes 6-12 sessions over an 8-week period with a Link Worker who will work with individuals, on a one-to-one basis, to understand ‘what matters to them’ and connect them with local sources of support. Pupils in the control group will receive signposting to sources of support from school staff. Data will be collected at baseline, 3- and 6-month follow-up. Acceptability and feasibility will be assessed via participant recruitment and retention, and via qualitative interviews. Interviews will also explore barriers and facilitators to engagement and implementation and mechanisms of change. Primary and secondary outcomes will be completed to assess response and completeness, including measures of loneliness, mental health and wellbeing.

**Discussion:**

The INACT study will provide preliminary evidence of the feasibility and acceptability of both the research design and social prescribing intervention. Results will inform a planned future randomised trial.

**Trial registration:**

ClinicalTrials.gov NCT06656663.

## Introduction

The transition from childhood to adolescence (with adolescence comprising the period between 10–24 years of age) is a critical development period, with major physical, psychological, and social changes occurring [1]. During this time, social connections, including friendships, are known to be cornerstones of healthy adolescent development [2]. Without social and community connections, negative impacts can occur [2], including loneliness, which in turn triggers negative cycles of psychophysiological symptoms [3]. Loneliness is longitudinally associated with depression symptoms from childhood to adolescence [4] and can result in physical manifestations, such as poor sleep, changes to appetite, and headaches [5,6]. Young people (YP) who report loneliness are also at increased risk of cardiovascular disease and psychiatric disorders in adulthood [7,8]. It is theorised that prolonged loneliness adversely affects health via multiple mechanisms, including the dysregulation of physiological systems involved in social connections [9].

Data from the UK show that YP report some of the highest prevalence rates of loneliness across different age groups, with 11.3% of YP aged 10-15 years old stating they always or often feel lonely [10]. This is not distributed equally, with higher levels of loneliness reported amongst younger individuals (14%), those who live in cities (19.5%) and those who receive free school meals (27.5%) [10]. In the last 30 years, data from international samples of YP suggests that levels of loneliness have been increasing, with marked rates of growth occurring from the late 1990’s onwards [11,12]. The Covid-19 pandemic further exacerbated feelings of loneliness, with 35% of YP reporting often feeling lonely during lockdowns [13].

A recent review and meta-analysis of interventions that tackle loneliness in YP demonstrated a moderate effect size overall [14]. However, most interventions targeted YP *at risk of loneliness* (e.g., due to physical health difficulties), rather than those *who reported lonelines*s. Effect sizes did not significantly differ based on intervention characteristics. However, the authors noted that a ‘one-size fits all’ approach is unlikely to reduce loneliness among YP, and that a tailored approach based on individual needs should be considered in future interventions. The authors of the review also noted evidence gaps including the lack of longer-term follow-up and targeted interventions for YP reporting loneliness, and the need to consider socioeconomic and other risk factors for targeted interventions [14].

Social prescribing (SP) is a mechanism of care aimed to address health inequalities by linking patients with non-medical forms of existing support within their local communities [15]. It usually involves a health or social care professional referring a patient to a Link Worker (LW), who co-develops a non-clinical plan with the patient based on their values, needs and preferences and connects them with existing community organisations to improve health, wellbeing or other aspects of the patient’s life [16]. Community support can include (but is not limited to) the arts, music, access to nature, volunteering, gardening, exercise, and broader support services [15]. The future priorities of the UK’s ‘Campaign to End Loneliness’ firmly support the need for SP services, highlighting the need for ‘connector services that reach, understand, and support lonely individuals’ to ‘increase connections, improve relationships and change an individual’s thinking’ [17]. These services can be delivered via psychological methods, one-to-one support, and group support. SP incorporates elements of all three [18].

Recent reviews into the impact of SP on loneliness suggest that SP can have a positive impact [19] and that both participants and service providers believe it to be helpful [20]. However, those studies were limited to adult populations and often lacked control groups [20]. When control groups are utilised, results suggest that SP is beneficial [21,22]. In one UK study on SP for adults who reported loneliness, 37% of those who received SP were classified as ‘not lonely’ at 3-month follow-up, compared to 20% of those in the matched control group [22]. There was also evidence to suggest that younger age groups (those ages 18-50 years old) benefitted more than older adults [22]. Similarly, a controlled trial in Australia exploring the impact of SP on 114 adults showed that 8 weeks after starting SP, the intervention group reported decreased loneliness with a large effect size [21]. Retention of those allocated to SP was also high at 79.4% [21]. Whilst the evidence for youth SP is less developed, a recent review found that SP is a promising way of tackling loneliness among YP, although there are methodological concerns, including the small number of participants, meaning robust conclusions could not be drawn [23]. Accordingly, there is a critical need for robust research that can provide insight into SP for YP who report loneliness.

SP is supposed to be an ‘all age’ model [24], yet research suggests that uptake from YP remains lower than for adults [25]. While YP see the benefits of SP [26], the dominant model of delivery through family doctors in the UK may be inhibiting youth involvement as they do not feel comfortable accessing wellbeing support in this way [27]. Schools may be better placed to deliver wellbeing initiatives and targeted interventions for loneliness, as they serve as a universal point of access for YP [28]. Schools could act as a conduit to connect YP to community support and have started to be used as a venue to facilitate SP in the UK [29]. The building blocks for SP are already in place, with the rollout of specialist youth LWs across England and an increasing number of community organisations delivering activities suitable for youth SP. However, SP has not yet been properly embedded into schools, and educational stakeholders have been underrepresented in SP pathway development. More work needs to be undertaken to ensure adequate information sharing between agencies, such as schools and LWs [29]. To date, no studies have explored SP in school settings.

Given the high rates of loneliness among YP and the lack of interventions targeting loneliness in this cohort [14], this proposed study will focus on piloting a novel SP pathway in schools for pupils reporting low community connection or loneliness. The aims of this pilot study are to ensure the following:

i) The SP pathway and study design are feasible, acceptable and suitable;
ii) The proposed measurement framework is tested for feasibility, acceptability and suitability, and
iii) Quantitative data are collected to inform a better estimation of the required sample size for a full trial.

### Research questions (RQs)

***(RQi)*** Is the SP pathway for youth reporting low community connection or loneliness feasible, acceptable and suitable to stakeholders?

***(RQii)*** Is the study design, including measurement framework, feasible, acceptable and suitable to stakeholders?

***(RQiii)*** Are the assumptions in the power calculation (specifically the intraclass correlation coefficients) realistic to allow for adequate power to be able to test for differences?

## Materials and methods

### Design

This manuscript relates to the INACT pilot study protocol V1.0 05-02-24 and follows the SPIRIT checklist (see S1 Checklist). Any details of protocol amendments will be submitted to the journal after approval from UCL ethics committee. INACT will adopt a two-group (intervention vs. active control) parallel randomised design, with YP as the unit of randomisation. YP allocated to the intervention arm will receive SP, while those in the control arm will receive signposting from school staff to activities in their local area. Outcomes will be assessed at baseline (T0), 3 months after being allocated to SP or signposting (T1), and 6 months after being allocated to SP or signposting (T2).

### Setting

This study will involve primary and secondary schools in England. We aim to recruit our sample from mainstream schools across 3 cities: London, Leeds, and Manchester. These sites have been selected due to their (i) urbanicity, (ii) diversity in socio-economic status, and (iii) high levels of deprivation, which are known to affect community connection and loneliness [10].

### Participants

We aim to recruit a total of 12 schools (6 primary schools and 6 secondary schools). In each primary school, one class from each of Years 4 and 5 (children aged 9 and 10 years old) will be selected, whilst in secondary schools, two classes each from Years 7 and 8 (children aged 12 and 13 years old) will be selected. Year 6 (children aged 11 years old) will be excluded from taking part because previous studies by the research team have shown that schools are reluctant to engage Year 6 students in research due to exam preparation [30,31]. Private schools will be excluded because they have greater resource to support YP with wellbeing difficulties and do not use unique pupil numbers linked to the National Pupil Database, which will be needed to explore factors associated with low community connection and loneliness.

Approximately 780 pupils (exact number dependant on class size) will complete baseline surveys to examine the prevalence rates of low community connection and loneliness and factors that predict this. Under realistic assumptions from other trials [31] and reviews on low community connection and loneliness [14] (low community connection/loneliness prevalence =  14%, participation rate =  71%), approximately 78 pupils will participate in the pilot study (39 receiving SP and 39 receiving signposting). YP eligible for the pilot study will be those reporting feelings of loneliness (≥6) using the 3 loneliness questions from the Good Childhood Index [32]. This corresponds to YP who report feeling lonely at least some of the time across all three questions. YP who are persistently absent from school will be included. YP reporting severe learning disability will be excluded.

In addition to YP, approximately 20 other stakeholders, namely LWs and school pastoral staff, will participate in the pilot study. Pre-intervention, a single member of staff in each participating school will be asked to complete a school wellbeing provision survey. Typically, this will be the named mental health lead; in schools where there is no named mental health lead, it may be the member of staff with primary responsibility for personal, social and health education (PSHE) provision or pastoral support. Post-intervention, school staff who were involved in signposting and LWs who provided SP will be asked to complete a feasibility, acceptability and suitability survey.

Finally, a subsample of YP (n =  24) and stakeholders (n =  18) in the intervention arm will be asked to take part in a qualitative interview to better understand the feasibility and acceptability of the SP pathway, as well as barriers and facilitators to engagement and implementation. Topic guides are shown in the S1 and S2 Files. Purposive sampling will be employed. For YP, purposive sampling will take into account socio-demographic factors, such as age, gender, ethnicity, site, loneliness score, and the degree to which they engaged in SP (none, some, a lot). For LWs and school staff, purposive sampling will take into account sociodemographic factors, location, role, and types of activities prescribed (for LWs). The number of interviews has been selected to be large enough to allow for adequate “information power” and to make meaningful comparisons between social prescribing pathway delivery and experiences in different sites [33] but not too large to dilute an in-depth rich analysis and exploration of individual participant accounts.

### Procedure

Our recruitment strategy will include (i) drawing on established school networks (e.g., the Schools in Mind network, containing over 30,000 schools), (ii) undertaking an extensive targeted social media campaign towards educational professionals in cities of interest; (iii) attending headteacher network meetings to promote the project; (iv) purchasing the Sprint education database, which contains up-to-date contact details on 570,000 teachers and senior staff at UK schools, (v) offering transparent and accessible communication regarding what school participation entails formalised in a Memorandum of Understanding (MOU), and (vi) compensation of £500 per school for administrative support.

Schools will be recruited by the methods specified above, beginning 01/03/24 and ending 30/06/24 with the aim of starting the pilot interventions on 03/01/25. Schools that express an interest will be asked to fill out a MOU agreeing to participate. As interest is likely to exceed capacity during the pilot, schools will be held in reserve (in case of drop out) or for participation in the full trial. Following recruitment of schools, YP in relevant year groups will be recruited in two stages. Firstly, schools will send letters to parents/carers of pupils in selected classes, including to those identified as persistently absent. The letter will be sent between 16/09/24-31/10/24 and provide information about the study and allow parents/carers to opt their children out of the study. Secondly, for parents and guardians that do not opt out their YP, the YP must assent by reading through an online information sheet and completing an online consent form before they can take part in the baseline survey. Data collection for baseline surveys and YP assent will start on 12/11/24 and be completed by 30/11/24 in all schools. Thus, participant recruitment will be completed by 30/11/24. Follow up data collection will be completed by 01/07/25 and the study completion date is the 31/08/25. For the qualitative interviews, separate information sheets and opt-in consent forms will be provided to parents/carers and YP who are interested in participating.

To identify eligible YP, baseline surveys will be administered to classes of pupils in relevant year groups during PSHE (or another appropriate lesson determined by the school). For pupils who are persistently absent from school, baseline surveys can be completed in their own time (e.g. at home), or with researchers or school pastoral staff at pupils’ convenience. Those who meet the inclusion criteria will then be randomly allocated to receive SP (the intervention) or signposting (the active control). Random allocation will be undertaken by a statistician at the pupil level, stratifying for location. Given the nature of the intervention blinding of participants or researchers is not possible. As LWs will only be able to work with individuals allocated to the intervention arm, this will prevent contamination effects, and all pupils will have access to the same list of community resources. Those allocated to SP will then be contacted by a LW who will arrange a time to meet with them. For those allocated to signposting, researchers will share the names of YP with school pastoral staff who will signpost them to community resources. Any YP identified as having significant mental health issues on the Me & My Feelings questionnaire [34] will be highlighted to pastoral staff regardless of intervention group.

YP who receive SP or signposting will then be followed up at 3 and 6 months using the same measures in the baseline survey, with additional questions on what they thought of the intervention. Participants will receive a £10 voucher for completing follow up measures. Those who were allocated to receive SP or signposting but chose not to receive it will still be given the option of completing follow-up questionnaires for Intention-To-Treat analysis. Feasibility, acceptability, and suitability measures will be administered to school staff and LWs 6 months after the intervention begins. Qualitative interviews with YP will take place at least 3 months after they have received SP and interviews with school staff and LWs will take place at least 6 months after SP has been implemented in their school. Stakeholders will be approached by the research team, taking into account socio-demographic information, questionnaire scores and SP engagement to allow for purposeful sampling. All personal data will be kept on the UCL Data Safe Haven and will be stored securely under a unique numerical identifier. The UCL Data Safe Haven is a certified data transfer and storage system that meets the ISO27001 information security standard and conforms to NHS Digital’s Information Governance Toolkit.

All data collection will be completed by 31/07/25 and results are expected in September 2025.

### Intervention and control

SP is a person-centred approach to wellbeing involving the co-development of a non-clinical prescription, between an individual (in this case, YP) and LW, based on the perceived reasons for the referral and the YP’s values, needs and preferences [24]^.^ LWs have an excellent knowledge of their local areas, via community asset mapping and networking, allowing them to connect individuals with different types of support and activities.

The SP intervention used in this study will be ‘YES’: Youth Engagement in Social prescribing. YES has been developed to connect children and young people with forms of support in their communities to improve wellbeing. It has been used nationally, for example as part of the Wellbeing While Waiting programme which is implementing SP for YP on waiting lists for mental health treatment [35].

The SP intervention will range from 6-12 sessions over an 8-week period. Sessions may take place online, via phone call, or in person. As part of the SP process, LWs will draw on psychological skills such as motivational interviewing and behavioural activation [18] as well as employ problem solving and goal setting [36]. Following the identification of individual needs and preferences, the LW will discuss available local activities and informal sources of support with YP that best match their interests and could help them to feel more connected. Then, with the YP’s agreement, the LW will facilitate their engagement with these activities, which could be done via the LW making links with the organisation and/or accompanying the YP to the first session. All LWs will have undergone training [37] and receive supervision from an experienced mental health professional and/or senior LW.

YP in the control group will receive signposting to social and community activities in their local area. This will consist of school pastoral staff providing YP with a leaflet detailing information on the same local sources of support identified by the LW from asset mapping.

### INACT measures and outcomes

**Fig 1:**
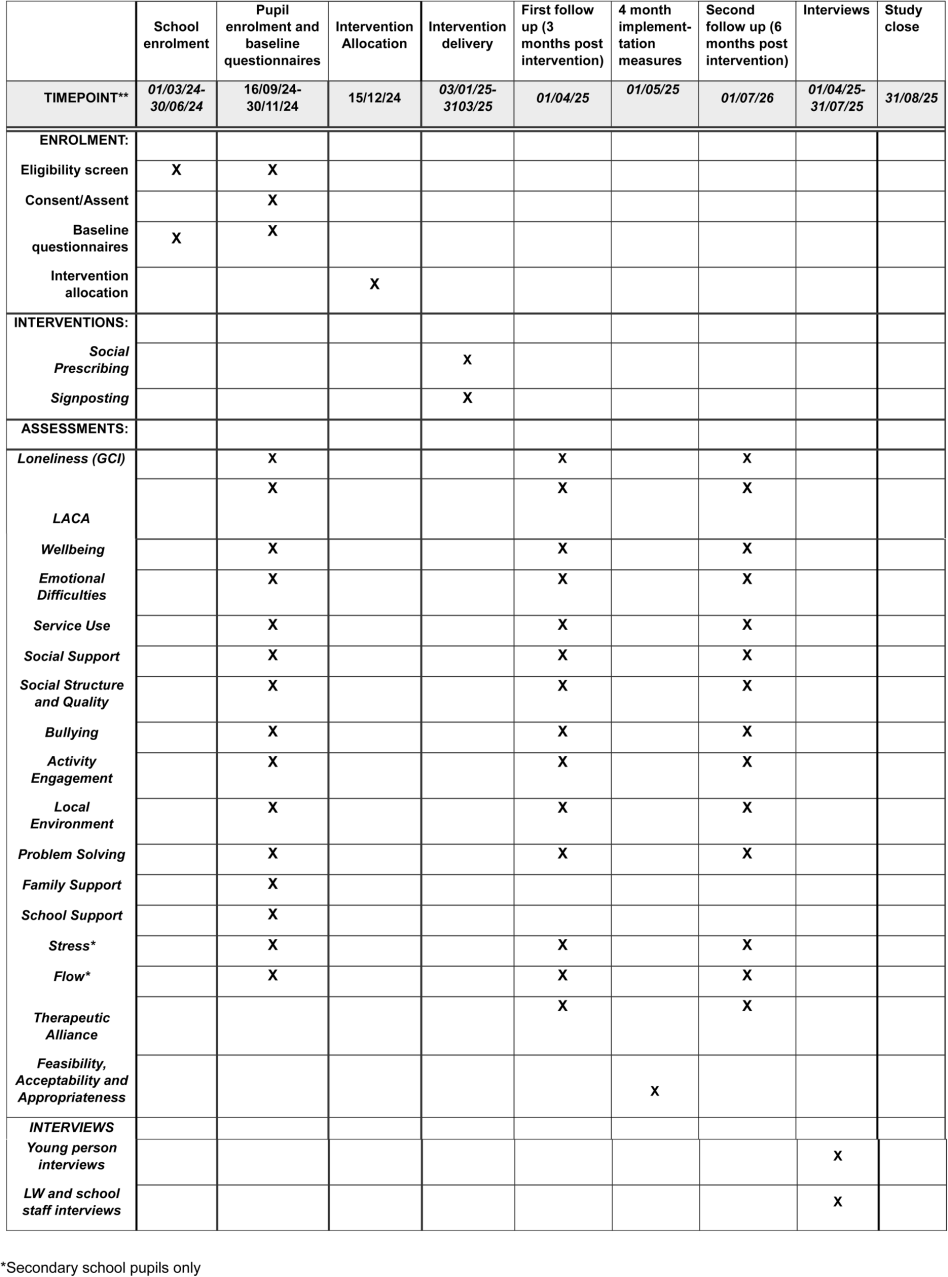
INACT Schedule of enrolment, interventions, and assessments.

A schedule of enrolment, interventions, and assessments is outlined in Fig 1.

#### SP implementation measures.

For school staff and LWs, feasibility, acceptability and appropriateness will be assessed with the Feasibility of Implementation Measure, Acceptability of Intervention Measure and Appropriateness of Intervention Measure [38]. Each questionnaire consists of 4 questions, each on a five-point Likert scale ranging from 1 ‘Completely Disagree’ to 5 ‘Completely Agree’. Higher scores indicate greater feasibility, acceptability, and appropriateness.

As the above measures are not validated to be used with YP, acceptability will also be assessed by the proportion of YP who take up SP after being offered it.

#### Study outcome measures.

The proposed primary outcome measure for INACT is the 3 loneliness questions from the Good Childhood Index [32]. Each question is scored on a three-point Likert scale ranging from 1 ‘hardly ever or never’ to 3 ‘often’. Higher total scores indicate higher levels of loneliness. This is assessed at baseline, 3 and 6 months. The primary timepoint is at 3 month follow up.

Secondary measures were chosen with young person and subject expert input. These include:

*For young people*

- Peer loneliness – assessed using the Loneliness and Aloneness Scale for Children and Adolescents (LACA) peer subscale [39]. This consists of 12 questions each on a four-point Likert scale ranging from 1 ‘never’ to 4 ‘always’. Higher total scores indicate higher peer loneliness. This is assessed at baseline, 3 and 6 months.
- Wellbeing – assessed using the Wellbeing Scale from the Kidscreen-52 [40]. This consists of 6 questions each on a five-point Likert scale ranging from 1 ‘not at all/never’ to 5 ‘extremely/always’. Higher total scores indicate greater well-being. This is assessed at baseline, 3 and 6 months.
- Emotional difficulties – assessed using the emotional difficulties subscale from the Me and My Feelings questionnaire [34]. This consists of 10 questions each on a three-point Likert scale ranging from 0 ‘never’ to 2 ‘always’. Higher total scores indicate greater emotional difficulties. This is assessed at baseline, 3 and 6 months.
- Service use – assessed using the Client Service Receipt of Inventory [41]. It consists of 11 questions asking about service use. Of these, 9 questions are about different types of service use and each are answered using a five-point Likert scale ranging between 1 ‘not at all’ to 5 ‘several times a week’. Each item is individually scored, and higher scores indicate more engagement with services. Two further questions ask about pupils accessing school wellbeing support and if they have missed school because of mental health/wellbeing difficulties. This is assessed at baseline, 3 and 6 months.
- Social Support – assessed using the Child and Youth Resilience Measure [42]. This contains 4 questions each question on a five-point Likert scale ranging from 1 ‘not at all’ to 5 ‘A lot’. Higher scores indicate greater social support. This is assessed at baseline, 3 and 6 months.
- Social structure and quality using questions adapted from the OECD Programme for International Student Assessment (PISA) 2022 [43] and Millenium Cohort Study 5 [44]. It consists of 5 questions asking about social support and quality. Of these, 4 questions each assess social structure and quality using a five-point Likert Scale ranging from 0 ‘I do not have any friends’ to 4 ‘every day or several times a day/a lot’. Higher scores indicate greater social structure and quality connections. The other question requires a numerical input on how many close friends the YP has. This is assessed at baseline, 3 and 6 months.
- Bullying – assessed using the bullying subscale from the Kidscreen-52 [40] for primary school pupils and the bullying subscale from the BeeWell survey [45] for secondary school pupils. For the Kidscreen-52 [40] this consists of three questions each on a five-point Likert scale, ranging from 1 ‘never’ to 5 ‘always’. For the BeeWell survey [45] this contains 3 items each on a four-point Likert scale ranging from ‘0” not bullied at all’ to 3 ‘a lot’. In both instances, higher scores indicate higher levels of bullying. This is assessed at baseline, 3 and 6 months.
- Activity engagement - assessed using the BeeWell survey [45]. It consists of 11 questions on activity engagement each on a six-point Likert scale ranging from 1 ‘never or almost never’ to 6 ‘most days’. Each item can be individually scored, or a higher overall score indicates more frequent engagement in multiple activities. This is assessed at baseline, 3 and 6 months.
- Local environment – assessed using questions from the Health Behaviour in School-aged Children (HBSC) 2022 survey [46]. It contains 4 questions each on a five-point Likert scale ranging from 1 ‘strongly disagree’ to 5 ‘strongly agree’. Higher scores indicate more positive views of ones environment. This is assessed at baseline, 3 and 6 months.
- Problem solving – assessed using the problem solving subscale from the Student Resilience Survey [47]. It consists of 3 questions each on a five-point Likert scale ranging from 1 ‘never’ to 5 ‘always’. Higher scores indicate better problem solving. This is assessed at baseline, 3 and 6 months.
- Family support – assessed using the family support subscale from the Student Resilience Survey [47]. It consists of 4 questions each on a five-point Likert scale ranging from 1 ‘never’ to 5 ‘always’. Higher scores indicate higher family support. This is assessed at baseline only.
- School support – assessed using the school support subscale from the Student Resilience Survey [47]. It consists of 4 questions each on a five-point Likert scale ranging from 1 ‘never’ to 5 ‘always’. Higher scores indicate higher school support. This is assessed at baseline only.
- Stress - assessed using the Perceived Stress Scale 4 [48]. This consists of 4 questions each on a five-point Likert scale ranging from 0 ‘never’ to 5 ‘very often’. Higher scores indicate higher levels of stress. This is assessed at baseline, 3 and 6 months. Due to its reading age, this is only being used with secondary school pupils.
- Flow - assessed using the General Flow Proneness Scale [49]. It consists of 13 items each on a five-point Likert scale each ranging from 1 ‘strongly disagree’ to 5 ‘strongly agree’. Higher scores indicate a higher proneness for flow-state experiences. This is assessed at baseline, 3 and 6 months. Due to its reading age, this is only being used with secondary school pupils.
- Therapeutic alliance – assessed using the Session Feedback Questionnaire [50]. It consists of four items each on a five-point Likert scale each ranging from 1 ‘not at all’ to 5 ‘totally’. Higher scores indicate higher therapeutic alliance. This is assessed at 3 and 6 months for those in the intervention (SP) group.

### Data analysis

***(Rqi)*** To assess the feasibility, acceptability and suitability of the SP pathway, descriptive statistics will be calculated from stakeholders’ questionnaire data responses, how many YP take up the SP offer, as well as via qualitative interview data which will be analysed using framework analysis [51] a method which allows for both deductive and inductive coding of transcripts across multiple stakeholder accounts.

***(Rqii)*** To assess the feasibility, acceptability and suitability of the study design, descriptive statistics will be calculated exploring measure completion rates, as well as from interview data using framework analysis [51].

Implementation data will be used to determine whether pathway refinements are needed and outcome measures will inform refinements to sample size (establishing ICCs and effect size). Crucially, the project will not proceed to full trial until pre-established “stop-go” criteria have been met (see Table 1). If the criteria are partially met, the research team will further develop trial plans (e.g., tweaking the SP pathway, reducing the number of follow-up measures, etc). This will be signed off by the Trial Steering Committee (TSC) prior to proceeding to full trial.

**Table 1:** Stop-go criteria.

Method	Indicator	Fully met (proceed)	Partially met (proceed with amendments agreed by TSC)	Not met (stop)
Acceptability of intervention	Qualitative reports of acceptability from young people, school staff and link workers	The majority (>50%) of stakeholders report the social prescribing intervention is acceptable	Some stakeholders report the social prescribing intervention is not acceptable but identify alterations that need to occur	The majority (>50%) of stakeholders report the social prescribing intervention is not acceptable even with modifications
	Proportion of young people attending an appointment with a link worker	55-100%	40-54%	0-39%
Acceptability of evaluation framework	Qualitative reports of acceptability from young people, school staff and link workers	The majority (>50%) of stakeholders report the social prescribing intervention is acceptable	Some stakeholders report the social prescribing intervention is not acceptable but identify alterations that need to occur	The majority (>50%) of stakeholders report the social prescribing intervention is not acceptable even with modifications
	Proportion of young people completing baseline measures	55-100%	40-54%	0-39%
Ability to collect 3-month data from pupils who completed baseline measures	Proportion of young people completing 3 month follow up	55-100%	40-54%	0-39%
Negative consequences of the intervention	Reported negative/adverse effects of social prescribing from young people, school staff or link workers	No evidence of substantially negative effects on young people	–	Evidence of substantially negative effects on young people

***(Rqiii)*** To assess whether power calculation assumptions are realistic, descriptive statistics, and point estimates of the within-person intraclass correlation coefficients (ICCs) for all instruments based on the pilot data will be calculated, as well as a bootstrap study exploring the upper bounds for the ICCs.

Where questionnaire data is missing from participants at random, multiple imputation will be employed. There will be no interim analysis given the short timescale (6 months) that participants are involved in the intervention.

### User involvement

User involvement is central to the INACT study. The research team has already worked with North Thames Applied Research Collaborative Patient and Public Involvement and Engagement (PPIE) group, as well as the Social Prescribing Youth Network’s Youth Advisory Group (YAG) to develop this protocol. Both groups were positive about SP and advocated for a ‘school-based’ approach over other options presented. There was interest from members of both groups in providing input into the INACT study, including reviewing study documents, interpretation of findings, co-authoring papers, as well as dissemination of results in creative and accessible formats (e.g., vlogs for YP). PPIE stakeholders will meet monthly at the start of the project to facilitate start up before moving to quarterly meetings.

### Ethical considerations

Ethical approval for this study has been obtained from the UCL Research Ethics Committee (6735/017). A copy of the ethics application is in the S3 File. As per similar studies [30,31], and in line with the UK Department for Educations’ procedures [52], opt out consent will be sought from parents/guardians, recognising their responsibilities to their YP, whilst maintaining YP autonomy through their assent. UCL Research Ethics Committee approved the full consent procedure, including the use of opt-out parental consent. Information Sheets in age-appropriate language will be co-developed in collaboration with the YAG. Participants will have the right to withdraw at any point, without needing to give a reason. Encrypted Dictaphones or secure videocall platforms will be used for all interviews. Questionnaire data will be anonymised and qualitative data will be pseudonymised. Personal data will be stored securely on the UCL Data Safe Haven conforming to NHS Digital’s Information Governance Toolkit.

### Monitoring adverse events

The ongoing conduct and progress of this study will be monitored by an independently chaired Data Monitoring Committee (DMC) and Trial Steering Group. The DMC will include a statistician, clinician and lay member of the public all of whom have no conflict of interest with INACT. The DMC is independent of the Sponsor and the charter is available on request from the corresponding author. The Steering Group will consist of academics, service users, clinicians and those involved in policymaking.

Monitoring of Adverse Events (AE), defined as a negative, emotional and behavioural occurrence, or sustained deterioration in a research participant, will be captured during INACT. This includes Serious Adverse Events (SAE) which are a threat to life: suicidal ideation, suicidal intent, hospitalisation due to psychiatric use of substances and death including suicide. Other AEs, such as violent behaviour, self-harm, physical injury, or any other event that an individual feels is important to report, will also be captured.

LWs and School Safeguarding Leads will judge whether they believe the AE is likely related to the intervention. On becoming aware of SAEs, the Principal Investigator/Deputy Trial Manager will report SAEs or AEs which are likely to be related to the intervention to University College London who is sponsoring the research, the DMC and Steering Committee. Other AEs will be collated and reported quarterly to these groups. School and research safeguarding protocols will also be followed as standard in addition to the reporting and documenting of AEs. Participants will have the right to stop participating in the intervention, and will be made aware of this by LWs and researchers when any AEs are reported. The study sponsor has insurance arrangements in place should participants be harmed as a result of the INACT trial.

## Discussion

Currently, there are several major gaps in research and implementation of interventions to increase social and community networks for YP. Interventions typically focus on YP perceived to be at risk of loneliness, rather than YP who report low community connection or loneliness. Studies also only report short-term outcomes and do not account for socio-demographic and other risk factors. Whilst specifically for SP, there are no studies which have tested impact using robust methodologies, such as Randomised Controlled Trials with YP. INACT will address these gaps by providing new information on how SP, which has been found to be beneficial in adult settings, can be implemented in schools to benefit YP reporting low community connection or loneliness. INACT will focus on major cities, which have higher rates of loneliness compared to towns and villages [10]. Moreover, the cities selected for INACT have high levels of deprivation where many YP are socially disadvantaged (e.g., experiencing poverty), which are further risk factors for loneliness [10]. This pilot study will help to identify any major barriers to implementation and address them before proceeding to full trial, road test the SP pathway, provide examples of implementation to share with LWs when implementing at scale, and confirm the suitability and robustness of outcome measures with YP.

The NHS Long term plan includes new approaches to supporting YP and bringing together partners in health, social care, education and the voluntary sector. INACT will create bridges between schools, LWs employed in health and social care settings, and voluntary organisations, to overcome the gaps in evidence in understanding what works in reducing loneliness in YP. Additionally, NHS England aims to triple the number of LWs to 9,000 over the next 10 years [53]. Findings from INACT will provide crucial guidance on how, and where, these LWs should be distributed to help tackle the pressing public health need of youth loneliness. If effective, INACT has the potential to lead to reductions in loneliness among YP and mitigate against both mental and physical ill health later in life, improve educational attainment and save health and social care resources. Findings will be communicated via the UCL Social Biobehavioural group website (https://sbbresearch.org/) as well as via conferences, academic papers and via schools and service user groups.

## Supporting information

S1 Checklist SPIRIT checklist. (DOC)

S1 File Topic guide for young people. (DOCX)

S2 File Topic guide for staff. (DOCX)

S3 File INACT ethics document. (DOCX)

## References

[1] Sawyer SM, Azzopardi PS, Wickremarathne D, Patton GC. The age of adolescence. Lancet Child Adolesc Health. 2018;2(3):223–8. doi: 10.1016/S2352-4642(18)30022-1 30169257

[2] Blum RW, Lai J, Martinez M, Jessee C. Adolescent connectedness: cornerstone for health and wellbeing. BMJ. 2022;379:e069213. doi: 10.1136/bmj-2021-069213 36302526 PMC9600165

[3] Matthews T, Danese A, Caspi A, Fisher HL, Goldman-Mellor S, Kepa A, et al. Lonely young adults in modern Britain: findings from an epidemiological cohort study. Psychol Med. 2019;49(2):268–77. doi: 10.1017/S0033291718000788 29684289 PMC6076992

[4] Qualter P, Brown SL, Munn P, Rotenberg KJ. Childhood loneliness as a predictor of adolescent depressive symptoms: an 8-year longitudinal study. Eur Child Adolesc Psychiatry. 2010;19(6):493–501. doi: 10.1007/s00787-009-0059-y 19777287

[5] Løhre A, Lydersen S, Vatten LJ. Factors associated with internalizing or somatic symptoms in a cross-sectional study of school children in grades 1-10. Child Adolesc Psychiatry Ment Health. 2010;4:33. doi: 10.1186/1753-2000-4-33 21167024 PMC3019130

[6] Mahon NE, Yarcheski A, Yarcheski TJ. Loneliness and health-related variables in early adolescents: an extension. Psychol Rep. 2003;93(1):233–4. doi: 10.2466/pr0.2003.93.1.233 14563055

[7] Caspi A, Harrington H, Moffitt TE, Milne BJ, Poulton R. Socially isolated children 20 years later: risk of cardiovascular disease. Arch Pediatr Adolesc Med. 2006;160(8):805–11. doi: 10.1001/archpedi.160.8.805 16894079

[8] Xerxa Y, Rescorla LA, Shanahan L, Tiemeier H, Copeland WE. Childhood loneliness as a specific risk factor for adult psychiatric disorders. Psychol Med. 2023;53(1):227–35. doi: 10.1017/S0033291721001422 34120674 PMC9874978

[9] Hawkley LC, Cacioppo JT. Loneliness matters: a theoretical and empirical review of consequences and mechanisms. Ann Behav Med. 2010;40(2):218–27. doi: 10.1007/s12160-010-9210-8 20652462 PMC3874845

[10] Office for National Statistics. Children’s and young people’s experiences of loneliness: 2018. London; 2018.

[11] Myhr A, Anthun KS, Lillefjell M, Sund ER. Trends in socioeconomic inequalities in norwegian adolescents’ mental health from 2014 to 2018: a repeated cross-sectional study. Front Psychol. 2020;11:1472. doi: 10.3389/fpsyg.2020.01472 32733331 PMC7358281

[12] Madsen KR, Holstein BE, Damsgaard MT, Rayce SB, Jespersen LN, Due P. Trends in social inequality in loneliness among adolescents 1991-2014. J Public Health (Oxf). 2019;41(2):e133–40. doi: 10.1093/pubmed/fdy133 30053062

[13] Oxford ARC Study. Achieving resilience during COVID-19 weekly report 2. Oxford; 2020. Available: https://oxfordarcstudy.com/2020/05/20/weekly-report-2/

[14] Eccles AM, Qualter P. Review: alleviating loneliness in young people - a meta-analysis of interventions. Child Adolesc Ment Health. 2021;26(1):17–33. doi: 10.1111/camh.12389 32406165

[15] National Academy for Social Prescribing. What is Social Prescribing? In: National Academy for Social Prescribing, [Internet]. 2021 [cited 13 Aug 2023]. Available from: https://socialprescribingacademy.org.uk/what-is-social-prescribing/#:~:text=Social%20prescribing%20connects%20people%20to,on%20what%20works%20for%20them., https://www.england.nhs.uk/personalisedcare/workforce-and-training/social-prescribing-link-workers/#:~:text=Social%20prescribing%20link%20workers%20connect,housing%2C%20financial%20and%20welfare%20advice.

[16] NHS England. Social Prescribing Link Workers. NHS England, [Internet]. 2021 [cited 13 Aug 2023]. Available from: https://www.england.nhs.uk/personalisedcare/workforce-and-training/social-prescribing-link-workers/#:~:text=Social%20prescribing%20link%20workers%20connect,housing%2C%20financial%20and%20welfare%20advice.

[17] Jones D, Jopling K, Kharicha K. Loneliness beyond Covid-19: Learning Lessons of the Pandemic for a Less Lonely Future. London; 2021. Available from: https://www.campaigntoendloneliness.org/wp-content/uploads/Loneliness-beyond-Covid-19-July-2021.pdf

[18] NHS England. Social prescribing: Reference guide and technical annex for primary care networks. NHS England; 2021.

[19] Bickerdike L, Booth A, Wilson PM, Farley K, Wright K. Social prescribing: less rhetoric and more reality. A systematic review of the evidence. BMJ Open. 2017;7(4):e013384. doi: 10.1136/bmjopen-2016-013384 28389486 PMC5558801

[20] Reinhardt GY, Vidovic D, Hammerton C. Understanding loneliness: a systematic review of the impact of social prescribing initiatives on loneliness. Perspect Public Health. 2021;141(4):204–13. doi: 10.1177/1757913920967040 34159848 PMC8295963

[21] Dingle G, Sharman LS, Hayes S, Johnson T. A controlled evaluation of social prescribing on loneliness for adults in Queensland: 8-week outcomes. Research Square (pre-print). 2023 [cited 13 Aug 2023]. Available from: https://www.researchsquare.com/article/rs-2853260/v1 10.3389/fpsyg.2024.1359855 PMC11049426 38680281

[22] Foster A, Thompson J, Holding E, Ariss S, Mukuria C, Jacques R, et al. Impact of social prescribing to address loneliness: A mixed methods evaluation of a national social prescribing programme. Health Soc Care Community. 2021;29(5):1439–49. doi: 10.1111/hsc.13200 33084083

[23] Hayes D, Jarvis-Beesley P, Mitchell D, Polley M, Husk K, [On behalf of the NASP Academic Partners Collaborative]. The impact of social prescribing on children and young people’s mental health and wellbeing’. London; 2023. Available from: https://socialprescribingacademy.org.uk/media/lrif2emh/evidence-review-the-impact-of-social-prescribing-on-children-and-young-peoples-health-and-wellbeing.pdf

[24] NHS England. Social prescribing. NHS England, [Internet]; 2020 [cited 13 Aug 2023]. Available from: https://www.england.nhs.uk/personalisedcare/social-prescribing/#:~:text=Social%20prescribing%20is%20an%20all,who%20are%20lonely%20or%20isolated

[25] Cartwright L, Burns L, Akinyemi O, Carder-Gilbert H, Tierney S, Elston J, et al. Who is and isn’t being referred to social prescribing? London; 2022.

[26] Olsson A. “Expanding their world”: Children and young people’s views of social prescribing as an approach to improving mental health. University College London; 2021.

[27] Young Minds & The Children’s Society. First port of call: The role of GPs in early support for young people’s mental health. London; 2021.

[28] Aviles AM, Anderson TR, Davila ER. Child and Adolescent Social-Emotional Development Within the Context of School. Child Adolesc Ment Health. 2006;11(1):32–9. doi: 10.1111/j.1475-3588.2005.00365.x 32811063

[29] Polley P, Hayes D, Husk K. National Survey of Children and Young People’s Social Prescribing in England; 2022.

[30] Hayes D, Moore A, Stapley E, Humphrey N, Mansfield R, Santos J, et al. School-based intervention study examining approaches for well-being and mental health literacy of pupils in Year 9 in England: study protocol for a multischool, parallel group cluster randomised controlled trial (AWARE). BMJ Open. 2019;9(8):e029044. doi: 10.1136/bmjopen-2019-029044 31481370 PMC6731836

[31] Hayes D, Moore A, Stapley E, Humphrey N, Mansfield R, Santos J, et al. Promoting mental health and well-being in schools: examining mindfulness, relaxation and strategies for safety and well-being in English primary and secondary schools-study protocol for a multi-school, cluster randomised controlled trial (INSPIRE). Trials. 2023;24(1):220. doi: 10.1186/s13063-023-07238-8 36959662 PMC10034911

[32] The Children’s Society. Loneliness in childhood: Exploring loneliness and well-being among 10–17 year olds. London; 2019.

[33] Malterud K, Siersma VD, Guassora AD. Sample Size in Qualitative Interview Studies: Guided by Information Power. Qual Health Res. 2016;26(13):1753–60. doi: 10.1177/1049732315617444 26613970

[34] Deighton J, Tymms P, Vostanis P, Belsky J, Fonagy P, Brown A, et al. The Development of a School-Based Measure of Child Mental Health. J Psychoeduc Assess. 2013;31(3):247–57. doi: 10.1177/0734282912465570 25076806 PMC4107815

[35] Fancourt D, Burton A, Bu F, Deighton J, Turner R, Wright J, et al. Wellbeing while waiting evaluating social prescribing in CAMHS: study protocol for a hybrid type II implementation-effectiveness study. BMC Psychiatry. 2023;23(1):328. doi: 10.1186/s12888-023-04758-0 37165351 PMC10170442

[36] Cooper M, Avery L, Scott J, Ashley K, Jordan C, Errington L, et al. Effectiveness and active ingredients of social prescribing interventions targeting mental health: a systematic review. BMJ Open. 2022;12(7):e060214. doi: 10.1136/bmjopen-2021-060214 35879011 PMC9328101

[37] NHS England. Social Prescribing Link Workers: Resource to support and embed social prescribing link workers. NHS England [Internet]. 2021 [cited 13 Aug 2023]. Available from: https://www.england.nhs.uk/personalisedcare/workforce-and-training/social-prescribing-link-workers/

[38] Weiner BJ, Lewis CC, Stanick C, Powell BJ, Dorsey CN, Clary AS, et al. Psychometric assessment of three newly developed implementation outcome measures. Implement Sci. 2017;12(1):108. doi: 10.1186/s13012-017-0635-3 28851459 PMC5576104

[39] Goossens L, Maes M. Loneliness and Aloneness Scale for Children and Adolescents (LACA). Encyclopedia of Personality and Individual Differences. Cham: Springer International Publishing; 2017. p. 1–5.

[40] Ravens-Sieberer U, Gosch A, Rajmil L, Erhart M, Bruil J, Duer W, et al. KIDSCREEN-52 quality-of-life measure for children and adolescents. Expert Rev Pharmacoecon Outcomes Res. 2005;5(3):353–64. doi: 10.1586/14737167.5.3.353 19807604

[41] Beecham J, Knapp M. Costing psychiatric interventions. Measuring mental health needs. London: Royal College of Psychiatrists; 2001. p. 200–24.

[42] Ungar M, Liebenberg L. Assessing Resilience Across Cultures Using Mixed Methods: Construction of the Child and Youth Resilience Measure. Journal of Mixed Methods Research. 2011;5(2):126–49. doi: 10.1177/1558689811400607

[43] PISA 2022 Assessment and Analytical Framework. OECD; 2023.

[44] UCL Social Research Institute C for LStudies. MCS Age 11 Child Self-Completion Questionnaire: England. London; 2011. Available from: https://cls.ucl.ac.uk/cls-studies/millennium-cohort-study/mcs-age-11-sweep/.

[45] University of Manchester. BeeWell Survey. University of Manchester [Internet]. 2024 [cited 18 Oct 2024]. Available from: https://beewellprogramme.org/wp-content/uploads/2024/09/2024-Update-GM-BeeWell-Survey.pdf

[46] Hulbert S, Eida T, Ferris E, Hrytsenko V, Kendall S. HBSC England National Report: Findings from the 2021-2022 HBSC study for England. Canterbur; 2023.

[47] Lereya ST, Humphrey N, Patalay P, Wolpert M, Böhnke JR, Macdougall A, et al. The student resilience survey: psychometric validation and associations with mental health. Child Adolesc Psychiatry Ment Health. 2016;10 44. doi: 10.1186/s13034-016-0132-5 27822304 PMC5093941

[48] Demkowicz O, Panayiotou M, Ashworth E, Humphrey N, Deighton J. The Factor Structure of the 4-Item Perceived Stress Scale in English Adolescents. European Journal of Psychological Assessment. 2020;36(5):913–7. doi: 10.1027/1015-5759/a000562

[49] Elnes M, Sigmundsson H. The General Flow Proneness Scale: Aspects of Reliability and Validity of a New 13-Item Scale Assessing Flow. Sage Open. 2023;13(1):. doi: 10.1177/21582440231153850

[50] Evidence Based Practice Unit. Session Feedback Questionnaire. London; 2012. Available from: https://www.corc.uk.net/media/1405/sfq_questionnaire.pdf

[51] Parkinson S, Eatough V, Holmes J, Stapley E, Midgley N. Framework analysis: a worked example of a study exploring young people’s experiences of depression. Qualitative Research in Psychology. 2015;13(2):109–29. doi: 10.1080/14780887.2015.1119228

[52] Department for Education. Research with children and young people. Department for Education [Internet]; 2021 [cited 30 Aug 2023]. Available from: https://user-research.education.gov.uk/-research-ethics/children-young-people.html#content

[53] NHS England. NHS Long Term Workforce Plan. London; 2023. Available from: https://www.england.nhs.uk/long-read/accessible-nhs-long-term-workforce-plan/

