# Supplementary material for: INcreasing Adolescent social and Community supporT (INACT): Pilot study protocol
Source: PLoS One. 2025 Mar 26;20(3):e0317823. doi: 10.1371/journal.pone.0317823 (PMC11940566; doi:10.1371/journal.pone.0317823)
Supplement: S3 File — (DOCX) [file pone.0317823.s004.docx]

**S4 INACT ethics document**

**note to applicants: it is important for you to include all relevant information about your research in this application form as your ethical approval will be based on this form. Therefore anything not included will not be part of any ethical approval.**

**You should read the Ethics Application Guidelines and have them available as you complete this form.**

APPLICATION FORM

| **SECTION A APPLICATION FOR ETHICAL REVIEW: HIGH RISK** |
| --- |

| \| **A1** \| \| --- \| | **Project Title:** INcreasing AdolesCent social neTworks and support (INACT) | |
| --- | --- | --- | --- |
|  | **Is this application for continuation of a research project that already has ethical approval? *For example, a preliminary/pilot study has been completed and this is an application for a follow-up project? If yes, please provide the information requested below.*** | |
|  | No |  |

| \| **A2** \| \| --- \| | **Principal Researcher**  *Please note that a student – undergraduate, postgraduate or research postgraduate cannot be the Principal Researcher for Ethics purposes.* | |
| --- | --- | --- | --- |
|  | Full Name: Dr Daniel Hayes | Position Held: Senior Research Fellow |
|  | Name and Address of Department:  Research Department of Behavioural Science and Health, Institute of Epidemiology & Health Care, University College London, 1-19 Torrington Place, London, WC1E 7HB | Email: |
|  |  | Telephone: |
|  |  | Fax: |
|  | **Declaration To be Signed by the Principal Researcher**   - I have met with and advised the student on the ethical aspects of this project design *(applicable only if the Principal Researcher is not also the Applicant).* - I understand that it is a UCL requirement for both students & staff researchers to undergo Disclosure and Barring Service (DBS) Checks when working in controlled or regulated activity with children, young people or vulnerable adults. The required DBS Check Disclosure Number(s) is: Dan Hayes 01818748761, Alex Burton 001830047413, Joely Wright 001838173511 - I have obtained approval from the UCL Data Protection Officer stating that the research project is compliant with the Data Protection Act 1998. My Data Protection Registration Number is: Z6364106/2024/02/87 - I am satisfied that the research complies with current professional, departmental and university guidelines including UCL’s Risk Assessment Procedures and insurance arrangements. - I undertake to complete and submit the ‘Continuing Review Approval Form’ on an annual basis to the UCL Research Ethics Committee. - I will ensure that changes in approved research protocols are reported promptly and are not initiated without approval by the UCL Research Ethics Committee, except when necessary to eliminate apparent immediate hazards to the participant. - I will ensure that all adverse or unforeseen problems arising from the research project are reported in a timely fashion to the UCL Research Ethics Committee. - I will undertake to provide notification when the study is complete and if it fails to start or is abandoned. | |

| **SIGNATURE:** | **DATE:** 07/02/24 |
| --- | --- |

| \| **A3** \| \| --- \| | **Applicant(s) Details** *(if Applicant is not the Principal Researcher e.g. student details):* | | | | |
| --- | --- | --- | --- | --- | --- | --- |
|  | Full Name: Prof Daisy Fancourt | | | | |
|  | Position Held: Professor of Psychobiology & Epidemiology | | | | |
|  | Name and Address of Department: Research Department of Behavioural Science and Health, Institute of Epidemiology & Health Care, University College London, 1-19 Torrington Place, London, WC1E 7HB | | | Email: | |
|  |  |  |  | Telephone: | |
|  |  |  |  | Fax: | |
|  | Full Name: Dr Alex Burton | | | | |
|  | Position Held: Senior Research Fellow | | | | |
|  | Name and Address of Department: Research Department of Behavioural Science and Health, Institute of Epidemiology & Health Care, University College London, 1-19 Torrington Place, London, WC1E 7HB | | | Email: | |
|  |  |  |  | Telephone: | |
|  |  |  |  | Fax: | |
| **Applicant(s) Details** *(if Applicant is not the Principal Researcher e.g. student details):* | | |  |  |  |
| Full Name: Emeline Han | | |  |  |  |
| Position Held: Research Fellow | | |  |  |  |
| Name and Address of Department: Research Department of Behavioural Science and Health, Institute of Epidemiology & Health Care, University College London, 1-19 Torrington Place, London, WC1E 7HB | Email: | |  |  |  |
|  | Telephone: | |  |  |  |
|  | Fax: | |  |  |  |
| Full Name: Joely Wright | | |  |  |  |
| Position Held: Research Assistant | | |  |  |  |
| Name and Address of Department: Research Department of Behavioural Science and Health, Institute of Epidemiology & Health Care, University College London, 1-19 Torrington Place, London, WC1E 7HB | Email: | |  |  |  |
|  | Telephone: | |  |  |  |
|  | Fax: | |  |  |  |
| \| **A4** \| \| --- \| | **Sponsor/ Other Organisations Involved and Funding** | | | | |
|  | 1. **Sponsor:**  **UCL**  **Other institution**   If your project is sponsored by an institution other than UCL please provide details:   1. **Other Organisations**: If your study involves another organisation, please provide details. *Evidence that the relevant authority has given permission should be attached or confirmation provided that this will be available upon request.*   The University of Manchester – Prof.’s Neil Humphrey and Pam Qualter are providing advice on (i) social/community connection and loneliness and (ii) school-based research design. They will not have access to identifiable participant data.  The Social Prescribing Youth Network have developed YES – the social prescribing package which is being investigated in WP3 in schools. They will not have access to participant data.   1. **Funding:** What are the sources of funding for this study and will the study result in financial payment or payment in kind to the department or College? *If study is funded solely by UCL this should be stated, the section should not be left blank.* This study is being funded by the Kavli Trust. | | | | |

| \| **A5** \| \| --- \| | **Signature of Head of Department [or Chair of your Departmental Research Ethics Committee]** *(This must not be the same signature as the Principal Researcher)* |
| --- | --- | --- |
|  | 1. **I have discussed this project with the principal researcher who is suitably qualified to carry out this research and I approve it.**   **I am satisfied that *[please highlight as appropriate]:***   1. **Data Protection registration:**  - **has been satisfactorily completed** - **has been initiated** - **is not required**  1. **a risk assessment:**  - **has been satisfactorily completed** - **has been initiated**  1. **appropriate insurance arrangements are in place and appropriate sponsorship [funding] has been approved and is in place to complete the study.**  **Yes  No** 2. **a Disclosure and Barring Service check(s):**  - **has been satisfactorily completed** - **has been initiated** - **is not required**   *Links to details of UCL's policies on the above can be found at:* [*http://ethics.grad.ucl.ac.uk/procedures.php*](http://ethics.grad.ucl.ac.uk/procedures.php)  ****If any of the above checks are not required please clarify why below.** |
|  |  |

| **PRINT NAME:** Prof Andrew Steptoe | |
| --- | --- |
| **SIGNATURE:** | **DATE:** 06/02/24 |

| **SECTION B DETAILS OF THE PROJECT** |
| --- |

****It is essential that Sections B1 and B2 are completed in simple understandable lay language that a non-expert could understand or you risk your project being rejected**

| \| **B1** \| \| --- \| | **Please provide a brief summary of the project in simple lay person’s prose outlining the intended value of the project, giving necessary scientific background.**  *(max 500 words)***.**  The transition from childhood to adolescence (with adolescence comprising the period between 10–24 years of age) is a critical development period, with major physical, psychological and social changes occuring. During this time, social networks and friendships are known to be cornerstones of healthy adolescent development. Without social and community connections, negative impacts can occur, including loneliness, which in turn triggers negative cycles of psychophysiological symptoms. Young people who lack community connection are more likely to be depressed and have issues such as poor sleep, low appetite and headaches. If not tackled, a lack of social and community connections and loneliness, can lead to poorer outcomes in adulthood, including psychiatric disorders and cardiovascular disease.  Recent reviews into interventions that tackle low social connection and loneliness suggest that benefits may be gained from young people engaging in community activities, hobbies and skills engagement. Many such activities are available within local communities, yet young people often do not know about them or how to access them. PPI work with young people, parents, and school representatives has outlined that schools maybe a good conduit to help young people engage in community activities, and that these may be completed via school staff, or other individuals such as social prescribers. The aim of INACT is to better understand pupils low community connection and loneliness, as well as ways which could improve it, via three aims:   1. Understand the current provision in schools to address low community connection and loneliness. 2. Understand the prevalence rates of young people who report low community connection and loneliness, as well as factors which affect this. 3. Pilot approaches to connect young people with community support sources when they report low community connection or feeling lonely. |
| --- | --- | --- |

| \| **B2** \| \| --- \| | **Briefly characterise in simple lay person’s prose the research protocol, type of procedure and/or research methodology (e.g. observational, survey research, experimental). Give details of any samples or measurements to be taken** *(max 500 words).*  INACT seeks to better understand (i) schools provision for community connection and loneliness, (ii) pupils low community connection and loneliness and factors which affect this, and (iii) ways to support pupils with low community connection and loneliness.  These three aims will be centralised around piloting a packaged social prescribing intervention (‘YES’: Youth Engagement in Social prescribing). YES has been developed by the Social Prescribing Youth Network (SPYN) to connect children and young people with forms of support in their communities to improve wellbeing. It has been used nationally (for example, as part of the Wellbeing While Waiting programme) and internationally (for example in Canada and Portugal), as well as across different settings (e.g. Colleges, Child Mental Health Services, Primary Care, and in the third sector). Social Prescribing has been found to positively impact mental health and wellbeing, and no negative outcomes have been reported. For INACT, YES will be piloted in primary and secondary schools to support pupils with low community connection and loneliness.  To achieve the aims specified above, INACT will answer the following questions:   1. What current provision do schools have in place to address low community connection and loneliness in pupils? 2. What are the prevalence rates of pupils who report low community connection and loneliness and what factors affect this? 3. What are stakeholder views on YES to address low community connection and loneliness in pupils. Do they perceive it to be feasible, acceptable and suitable and is there any evidence of impact?   Each question will form a separate work package (WP) for INACT.  ***WP1, Question (i: current provision) methodology and analysis:***  When they sign up for INACT, schools will be asked to complete a survey on what provision they have for community connection and loneliness. The survey will ask about form (e.g. universal, targeted/indicated), function (e.g. environmental, developmental, informational), intervention agent (e.g. school staff, external professionals), and evidential basis (e.g. new and untested, local evaluation, national/international evidence base). Data will be analysed descriptively and using content analysis (for any open text boxes) to explore current school provision for low community connection and loneliness.  ***WP2 Question (ii prevalence rates and predictors), methodology and analysis:***  Once schools have signed up for INACT, classes of pupils will be selected by schools in the Autumn term of 2024 to complete a survey to understand the prevalence rates and factors associated with low community connection and loneliness. This will include the following questionnaires (see appendix for references for validated measures and the questions being asked for non-validated measures)   - *Socio-demographic information - Loneliness: Loneliness and Aloneness Scale for Children and Adolescents (LACA) – Peer Subscale (Marcoen, Goossens, & Caes, 1987) - Direct Loneliness: Good Childhood Index Survey 2020. - Peer Support: Child and Youth resilience measure. Peer support subscale (Ungar & Liebenberg, 2011). - Peer social structure and quality (adapted from PISA 2022 and MCS4) - Family support: Student Resilience Survey (SRS) – Family support subscale (Sun & Stewart, 2007) - School support: Student Resilience Survey (SRS) – School support subscale (Sun & Stewart, 2007) - Problem solving: Student Resilience Survey (SRS) – Problem solving subscale (Sun & Stewart, 2007) - Emotional difficulties: Me & My Feelings questionnaire - Emotional difficulties subscale (Deighton et al., 2012) - Wellbeing: Kidscreen 52 – Wellbeing subscale (Ravens-Sieberer · 2008) - Problem solving Kidscreen 52 - Problem solving subscale (Ravens-Sieberer · 2008) - Bullying: Kidscreen 52 - Bullying subscale (Ravens-Sieberer · 2008) - *Engagement in activities: Beewell survey, 2022 - Short Client Service Receipt of Intervention (Beecham and Knapp, 2001) - *Local environment (adapted from HBSC 2022 survey).   Additionally for secondary schools, the following measures will be used (note these have not been used for primary school pupils due to readability)   - Stress - Perceived Stress Scale 4 (Demkowicz, 2019) - Flow - Flow Proneness Scale (Elnes, 2023) - *Bullying: Beewell survey, 2022 – to be used instead of the Kidcsreen 52 in primary scools   *non-validated measures  Data will be analysed descriptively (to explore prevalence rates), as well as using regression models (such as multi-level modelling) to explore predictors of low social connection and loneliness.  ***WP3 Question (iii piloting YES social prescribing), methodology and analysis:***  Once schools are ready to implement YES (Autumn 2024) it will be piloted. Pupils that have been identified as having low community connection or loneliness (using the survey data from WP2, question ii), will be randomly allocated to receive further support (either signposting, or YES). Upon random allocation, schools will inform pupils (and their parents/guardians) of the support they can receive. Both interventions are outlined further below:   - YES (social prescribing) is a person-centred approach to wellbeing involving the co- development of a non-clinical prescription, between a young person and Link Worker/social prescriber, based on the perceived difficulties and the young persons values, needs and preferences. Link Workers have an excellent knowledge of their local areas, via community asset mapping and networking, allowing them to connect individuals with different types of available support and activities. Typically, social prescribing ranges from 6-12 sessions (average 8 sessions) over an 8-week period. Sessions may take place online, via phone call, or in person. - Signposting is an approach where an individual (in this instance school staff) provides a young person with sources of support in their local communities that they may be interested in participating in. Signposting tackles individuals’ lack of understanding on what is available and provides them with information on how to access the community support, in leaflet form, so they are able to take it home. Signposting is often 1 session and may take place online, via phone call, or in person.   Those that take up the offer they were allocated (signposting or YES) will then be followed up at three and six months using the same survey as outlined in WP2, question ii, with additional questions on what they thought of the intervention and therapeutic alliance with the link worker (Therapeutic Alliance (Session Feedback Questionnaire, EBPU, 2012) for those in the social prescribing arm. Those that do not take up the offer, will still be given the option of completing follow up questionnaires for Intention-To-Treat analysis (however, as outlined in the information sheets, this is optional and not include measures of therapeutic alliance).  Other data collected during piloting will include:   1. An implementation survey with Link workers and school pastoral staff. This will be collected 6 months after the start of YES and signposting. This is a validated 12-item scale, assessing the acceptability, appropriateness and feasibility of the interventions (Weiner et al., 2017). Each construct consists of four items on a five point Likert scale (1 = completely disagree to 5 = completely agree). Higher scores indicate a more favourable appraisal of the intervention. Link Workers and school pastoral staff will also be asked to complete data on the average number of sessions they had with a young person and the different types of contact (e.g. over the phone, face-to-face) 2. Qualitative data. This data will be used to explore the acceptability, suitability and feasibility of the interventions in depth. Individual interviews will be conducted with staff and Link Workers as well as interviews with young people depending on their availability and preference. Interviews will occur at least 3 months after the pupil has received any intervention. Interviews with staff will occur at least six months after the start of the intervention in their school.   Participants in interviews will be asked to complete an optional demographics questionnaire (attached), to capture the spread of different demographics in the sample and to enable sufficient description of the demographic make-up of the sample in reports of the findings (which is standard practice in peer-reviewed publications).  To assess the feasibility, acceptability and suitability descriptive statistics will be calculated from stakeholders’ questionnaire data (i.e. FIM/IAM/AIM), as well as via interview data using framework analysis, a qualitative method which allows both a priori issues and emergent data-driven themes to guide the analytic process. Any evidence of impact will be explored looking for changes in outcome measure scores prior and after those that received an intervention. |
| --- | --- | --- |
|  | *Attach any questionnaires, psychological tests, etc.* *(a standardised questionnaire does not need to be attached, but please provide the name and details of the questionnaire together with a published reference to its prior usage).* |

| \| **B3** \| \| --- \| | Please provide details of the potential participants for this project, including how they will be selected and recruited.  **School recruitment**  Schools will be made aware of INACT via newsletters and social media posts (e.g. via UCLs Social Biobehavioural group and UCL’s Institute of Epidemiology and Healthcare). Those that are interested in participating will contact the research team to find out more. We will recruit our sample from mainstream schools across 6 cities in the UK (final cities to be decided on depending on schools that sign up to INACT, but these will likely be: London and the surrounding area, Birmingham and the surrounding area, Manchester and the surrounding area, Liverpool and the surrounding area, Leeds and the surrounding area, and Sheffield and the surrounding area). Major cities and their surrounding areas are the focus of INACT due to their (i) urbanicity and (ii) diversity in socio-economic status and high levels of deprivation which are known to affect community connection and loneliness.  It is anticipated that a total of 12 schools will be recruited for INACT (6 primary schools and 6 secondary schools). In each primary school, one class from each of Years 4 and 5 will be selected to participate in INACT, whilst in secondary schools, two classes each from Years 7 and 8 will be selected. Private schools will be excluded as they have greater resource to support pupils with wellbeing difficulties and do not use unique pupil numbers which are linked to the National Pupil Database (which will be needed to explore factors which predict low community connection and loneliness (WP2).  **Staff recruitment**  ***WP1, question i (usual provision survey)***  Low community connection and loneliness school provision surveys will be completed by a single member of staff in each school. Typically, this will be the named mental health lead; in schools where there is no named mental health lead, it may be the member of staff with primary responsibility for personal, social and health education (PSHE) provision or pastoral support. Schools will be asked to identify this individual and provide their contact details when they sign up for INACT.  ***WP3, question iii (piloting social prescribing versus signposting)***  School staff providing signposting support and link workers in schools providing YES will be sent the feasibility, acceptability and suitability survey via the research team. Schools will be asked to provide contact details of these individuals when they sign up for INACT.  **Pupil recruitment**  For survey data for young people, opt out consent will be used when contacting parents. This process is the same as other approved trials by UCL ethics committee for school research (e.g. Education for Wellbeing: ethics number: 6735/009 and 6735/014). In line with GDPR, privacy notices are issued as part of the opt out process. All young people are under 16 and will require parental consent and will also need to consent to questionnaires prior to taking part.  ***WP2, question ii (prevalence rates and predictors)***  Following recruitment of schools for INACT, participants in relevant year groups will be recruited in two stages: First, schools will send letters to parents/carers of pupils in selected classes (1 class in each from Year 4 and Year 5 in primary schools and 2 classes from each in Years 7 and 8). The letter will provide information about INACT and explains parents/carers’ right to opt their child out. It will also explain that pupils will only be involved in the study if they consent before completing the survey. Second, pupils must consent; reading through an online information sheet and ticking boxes agreeing to take part. This will likely be in a lesson, such as PSHE, but this will be determined by the school. For individuals who are absent from school that day (e.g. due to illness), schools will be asked to make provision for pupils to complete this, at another time. Pupils, or parents or pupils, who do not consent/consent to take part will engage in an activity suggested by the school (such as quiet reading) while others complete the survey.  ***WP3, question iii (piloting YES versus signposting)***  Pupils that meet the threshold for low community connection or loneliness, as identified by their scores on the previous questionnaire, will then be randomly allocated to receive YES or signposting by the research team. Schools will be informed of this and inform parents and pupils that they have been allocated to receive an intervention. Those that want to take it up will then meet with a member of the school pastoral staff (for signposting) or a school link worker (for YES).  **Further information**  It will be made clear to all participants that they have no obligation to take part in any part of INACT (i.e. their participation in the study is completely voluntary) and that they can withdraw at any time. As all young people are aged under 16, parental consent will be sought, as well as the young person’s consent. The research is compliant with UK GDPR legislation and privacy notices will be issued with information sheets. |
| --- | --- | --- |

| \| **B4** \| \| --- \| | **Have collaborating departments whose resources will be needed been informed and agreed to participate?**  *Attach any relevant correspondence.*  University College London, SPYN, and the University of Manchester are clear in their roles for the project and have worked together on other projects.  Collaborator emails attached. |
| --- | --- | --- |

| \| **B5** \| \| --- \| | **How will the results be disseminated, including communication of results with research participants?**  Results may be disseminated through a variety of outlets, including, but not limited to:   - Feedback to schools and the funder (Kavli Trust) - Conference presentations - Academic publications - Non-technical reports - UCL’s Social Biobehavioural Unit website: <https://sbbresearch.org/news-events/> |
| --- | --- | --- |

| \| **B6** \| \| --- \| | **Please outline any ethical issues that might arise from the proposed study and how they are be addressed.** *Please note that all research projects have some ethical considerations so do not leave this section blank.*  Obtaining consent:  Consent: Consent forms will make participants aware of their right to withdraw at any point during INACT. For survey data with young people, opt out consent will be used. As all young people are aged under 16, parent/guardians will be given the opportunity to exclude their children from the research project i.e. ‘opt out’. Additionally, young people will have to electronically consent to research prior to filling out the survey. The information sheet will make this clear, outlining that they do not have to start if they don’t want to, as well as that they can stop at any time. This process is the same as per other approved trials by UCL ethics committee for school research (e.g. Education for Wellbeing: ethics number: 6735/009 and 6735/014).  All other data will utilise opt in consent. For young people (all of whom are aged under 16) participating in qualitative data, parents will be given the opportunity to include their children in the research project via schools distributing qualitative information sheets detailing the study. Additionally, young people will consent to take part in the research prior to any qualitative data collection (e.g. interviews). The information sheet will make it clear that participation is voluntary, outlining to participants that they do not have to start if they don’t want to, as well as that they can stop at any time. Parents/guardians will be allowed to be present in a young persons interview, if this is requested by the young person. School staff and Link Workers will also have to consent prior to being involved in any data collection by researchers.  Protecting participants:  Confidentiality and anonymity:  A unique password will be generated for each young person, school staff member and link worker. In order to access surveys, participants will need to enter their password. Participants will not be required to fill out their names, date of birth, or any identifiable data. Instead, the unique password will allow any survey data to be matched (i.e. for those that take part in both WP2 and WP3). A unique ID will be created for each individual and a list of names and associated unique ID numbers will be held separately from the data files.  Identifiable data is only provided to the INACT research team all based within University College London. Other researchers will not have access to this. Schools send the data in an encrypted file to University College London and this data will be stored in the UCL Data Safe Haven. The container file itself (UCL Data Safe Haven) is certified to the ISO27001 information security standard and conforms to NHS Digital's Information Governance Toolkit and can only be accessed by approved members of the INACT team via a dual authentication factor (username and password + phone confirmation). At the end of INACT (September 2025) all identifiable survey data is destroyed.  All interview data will be treated as confidential between the participants and the research team (the participant will be informed of this at the outset). However, if participants raise any information that indicates that they or another person is at risk of serious harm during any part of INACT, then this information will be immediately passed on to the appropriate people (i.e. both UCL safeguarding team and the schools safeguarding lead) in order to safeguard them. Participants will also be informed of this at the outset. All interview transcripts will be de-identified and treated confidentially, with any identifying details spoken by the participants in their interviews, such as names and places, removed. Care will be taken in reports to ensure that any data is not traceable to a particular individual.  Interviewees will be given a unique identifier that we will use to label their audio files and transcripts. This identifier will be linked to their names (and contact details where necessary, e.g. telephone numbers for staff members where telephone interviews are preferred) throughout the duration of the study, which will be stored in the UCL Data Safe Haven. Audio recordings will be deleted at the end of the study (September 2025).  Protection from harm:  The surveys and interviews will be asking young people about their feelings around low community connection, loneliness, wellbeing and mental health, and if they found social prescribing acceptable. School staff and Link Workers will be asked about intervention feasibility, acceptability and suitability. None of the questions are intentionally designed to induce stress (e.g. as part of experimental design), nor is their evidence in the literature, or from similar studies (e.g. Education for Wellbeing ref. 6735/009 and 6735/014) that they do. However, we recognise that some participants may find answering questions stressful or upsetting. Everyone participating in the research, will be provided with information on who they can contact (e.g. school teacher or parent for young people and Samaritans or school staff and Link Workers) to get support and discuss any concerns they have. |
| --- | --- | --- |

| **SECTION C DETAILS OF PARTICIPANTS** |
| --- |

| \| **C1** \| \| --- \| | **Participants to be studied**   \| **C1a. Number of volunteers:** \| ~800 (dependant on class sizes) \| \| --- \| --- \| \| Upper age limit: \| 65 \| \| Lower age limit: \| 8 \|   **C1b. Please justify the age range and sample size:**  The upper age limit is to include school staff and link workers. The lower age is to include young people in year 4 (the lowest age group to participate in INACT).  Approximately 780 pupils (exact number dependant on class size) will complete surveys to identify the prevalence rates of low community connection and loneliness and factors which predict this (WP2).  For the INACT pilot (WP3), under realistic assumptions from other trials (e.g. Education for Wellbeing) and reviews on low community connection and loneliness (e.g. Eccles, 2020): low community connection/loneliness prevalence = 14%, participation rate=71%), approximately 78 pupils will participate in the pilot (39 receiving YES and 39 receiving signposting).  The additional 20 participants will consist of interviews/implementation data on feasibility/acceptability and suitability of social prescribing with school staff/Link Workers (WP3), and the low community connection/loneliness school provision survey (WP1). |
| --- | --- | --- | --- | --- | --- | --- | --- | --- |

| \| **C2** \| \| --- \| | **If you are using data or information held by a third party, please explain how you will obtain this. You should confirm that the information has been obtained in accordance with the UK Data Protection Act 1998.**  We will use information held by schools and provided to the research team (pupil name, pupil class, pupil number) in order to generate the unique pupil password for the survey.  For WP2 (prevalence rates and predictors), the research team will draw on NPD data (including pupils ethnicity, if they have English as a second language, if they receive free school meals, and postcode), which is held by the Department for Education and linked to the participants unique pupil number.  Information Sheets will outline to participants what data is being provided and the purposes it will be used for and this data will be obtained in accordance with the UK Data Protection Act 1998. |
| --- | --- | --- |

| \| **C3** \| \| --- \| | **Will the research include children or vulnerable adults such as individuals with**  **a learning disability or cognitive impairment or individuals in a dependent or unequal relationship?**  **Yes**  **No**    How will you ensure that participants in these groups are competent to give consent to take part in this study? *If you have relevant correspondence, please attach it.*  For WP2 and WP3, the study will involve children under the age of 16. We will ask parents to consent to their children and young people participating in INACT, as well as get consent from young people. Both parents and young people will receive information about INACT, including its aims, what will happen if they take part, and that participation is voluntary. Information sheets will be age appropriate. Researchers and school staff will be well placed to explain the information sheet to young people, and asses their understanding and competence to decide to participate. This approach is the same as in the Education for Wellbeing programme (UCL ethics ref. 6735/009 and 6735/014) as well as other projects such as HeadStart (UCL ethics ref 8097/003) and no problems or concerns occurred during this process. |
| --- | --- | --- |

| \| **C4** \| \| --- \| | **Will payment or any other incentive, such as gift service or free services, be made to any research participant?**  **Yes**  **No**    If yes, please specify the level of payment to be made and/or the source of the funds/gift/free service to be used.  WP1-WP3: Schools will be reimbursed for administration time up to a maximum of £500 per school for participating in INACT. Schools will invoice the research team for the full amount at the end of the study (September 2025) and this will be sent via bank transfer.  WP3: Young people who participate in the INACT pilot study will receive a £10 Love2Shop voucher reimbursement for survey completion at 3 and 6 month follow up as reimbursement for their time (maximum £20). Young people who take part in an interview will receive a £20 Love2Shop voucher as reimbursement for their time.  Please justify the payment/other incentive you intend to offer.  Schools: This amount is to cover the administration time associated with being part of INACT (e.g. sending out letters to parents, collating of pupil lists, administrating surveys in WP2). This is proportionate and in line with other school research projects (i.e. Education for Wellbeing programme: UCL ethics ref. 6735/009 and 6735/014)  Pupils: This is for reimbursement for their time. |
| --- | --- | --- |

| \| **C5** \| \| --- \| | **Recruitment**  (i) Describe how potential participants will be identified:  **School identification**  Schools will be made aware of INACT via newsletters and social media posts (e.g. via UCLs Social Biobehavioural group and UCL’s Institute of Epidemiology and Healthcare). Those that are interested in participating will contact the research team to find out more. We will recruit our sample from mainstream schools across 6 cities in England (final cities to be decided depending on who SPYN work with, but these will likely be: London and the surrounding area, Birmingham and the surrounding area, Manchester and the surrounding area, Liverpool and the surrounding area, Leeds and the surrounding area, and Sheffield and the surrounding area). Major cities and their surrounding areas are the focus of INACT due to their (i) urbanicity and (ii) diversity in socio-economic status and high levels of deprivation which are known to affect community connection and loneliness.  **Staff identification**  ***WP1, question i (usual provision survey)***  Schools will be asked to identify an individual to do this and provide their contact details when they sign up for INACT. Typically, this will be completed by the named mental health lead; in schools where there is no named mental health lead, it may be the member of staff with primary responsibility for personal, social and health education (PSHE) provision or a pastoral support staff member.  ***WP3, question iii (piloting YES versus signposting)***  Schools will be asked to identify the school pastoral staff member(s) and school link workers that will be involved in signposting when they sign up for INACT (and provide school staff member details once pathways have been developed).  **Pupil identification**  ***WP2, question ii (prevalence rates and predictors) and WP3, question iii (piloting YES versus signposting)***  Following recruitment of schools for INACT, schools will be asked to select classes of pupils to participate in INACT (1 class in each from Year 4 and Year 5 in primary schools and 2 classes from each in Years 7 and 8).  ***WP3, question iii (piloting YES versus signposting)***  Pupils that meet the threshold for low community connection or loneliness will be identified by their scores on the WP2 survey.  (ii) Describe how potential participants will be approached:  **Schools**  Schools that are interested in taking part in INACT will be asked to contact the research team expressing their interest.  **Staff being approached**  **Surveys and qualitative data**  ***WP1, question i (usual provision survey) and WP3, question iii (piloting YES versus signposting)***  After schools have provided details of these individuals to the INACT research team, they will be contacted by the researchers (i.e. via email) with further information about the study.  **Pupils being approached**  **Surveys**  ***WP2, question ii (prevalence rates) and WP3, question iii (piloting YES versus signposting)***  Once schools have been accepted into the project and they have identified relevant classes of pupils, letters will be sent out to parents/guardians of young people in selected classes outlining the study in more detail and asking parents to ‘opt out’ if they do not want their young person to be involved in the INACT prevalence survey or pilot study. For parents/guardians that choose not to opt out, information sheets will then be provided to those young people in class, either via the school staff member or the researcher.  **Qualitative data**  ***WP3, question iii (piloting YES versus signposting)***  Young people who were allocated to receive social prescribing or signposting will be contacted by a school staff member not involved in the intervention (e.g. their form tutor) and asked if they are interested in participating an in interview on their experiences. For those that are interested, schools will send a parent/guardian information sheet and consent form asking them to opt in to participate in an interview.  (iii) Describe how participants will be recruited:  **School recruitment**  Schools that (i) fall within, or close to a major city and (ii) are willing to participate in INACT will be accepted into the study. If demand exceeds supply, we will operate a first come-first served basis, with additional schools being put on a wait list.  **Staff recruitment**  **Surveys and qualitative data**  ***WP1, question i (usual provision survey) and WP3, question iii (piloting YES versus signposting)***  After participants have received the information sheet, they will be given a chance to think about the study and to ask questions to the researchers before making a decision about taking part. Consent will be obtained for those that want to participate.  **Pupil recruitment**  **Surveys**  ***WP2, question ii (prevalence rates and predictors) and WP3, question iii (piloting YES versus signposting)***  We are mirroring processes used in other school-based research projects approved by UCL ethics committee (e.g. Education for Wellbeing: ethics number: 6735/009 and 6735/014).  Parents/guardians of pupils taking part in the intervention will be sent information sheets via schools detailing the nature of the study and asked to opt out if they do not want their child to take part in INACT. The information sheet details researcher contact information if parents/carers have queries or questions. Parents/carers will be given 2 weeks to return opt out consent to researchers from when schools send out letters. They can do this by emailing the researchers. Eligible pupils (i.e. those whose parents did not opt out) will then be provided with online information sheets about the study (including the right not to take part) and asked to consent electronically via ticking a box at the bottom of the page. Those that do not tick the box will not be able to access the survey.  **Intervention allocation and follow up surveys**  ***WP3, question iii (piloting YES versus signposting)***  Pupils that are identified as meeting the threshold for low community connection and loneliness (on the WP2 survey) will be randomly allocated to YES or signposting by the research team. The research team will feed this information back to schools, who will in turn, offer the allocated intervention to pupils (informing their parents). For pupils who are allocated an intervention, schools will be asked to facilitate the completion of two follow up surveys at 3 and 6 months after the intervention began (containing the same questions as in WP2).  **Qualitative data**  ***WP3, question iii (piloting YES versus signposting)***  Young people who were allocated to receive social prescribing or signposting will be contacted by a school staff member not involved in the intervention (e.g. their form tutor) and asked if they are interested in participating an in interview on their experiences.. For those that say yes, schools will send out information sheets and consent forms to their parents/guardians (by email) which can be sent back directly to the research team or via the school. Both the parents/guardians and young people will be given a chance to think about the study and to ask the researchers questions before making a decision about them (or their young person) taking part. If both parent/guardian and young person consent/consent to participate, the researcher will arrange a time to meet with the young person over teams, in person, or phone for the interview  *Attach recruitment emails/adverts/webpages. A data protection disclaimer should be included in the text of such literature.* |
| --- | --- | --- |

| \| **C6** \| \| --- \| | **Will the participants participate on a fully voluntary basis?**  **Yes**  **No**  **Will UCL students be involved as participants in the research project?**  **Yes**  **No**  *If yes, care must be taken to ensure that they are recruited in such a way that they do not feel any obligation  to a teacher or member of staff to participate.*  **Please state how you will bring to the attention of the participants their right to withdraw from the study without penalty?**  Information sheets will be sent to all participants involved: school staff, link workers and young people (as well as the young persons parent/guardian). The right to withdraw is highlighted on these, as well as on consent forms. |
| --- | --- | --- |

| \| **C7** \| \| --- \| | **CONSENT**  **Please describe the process you will use when seeking and obtaining consent.**  **Survey data**  **Pupil survey data (WP2, question ii (prevalence rates and predictors) and WP3, question iii (piloting YES versus signposting)**  Parents/guardians of pupils will be sent information sheets via schools detailing the nature of the study and asked to opt out if they do not want their child to take part. The information sheet details researcher contact information if parents/guardians have queries or questions. Parents/carers will be given sufficient time to return opt out consent to researchers from when schools send out letters. Eligible pupils (e.g., those whose parents did not opt out) will then be provided with online information sheets about the study (including the right not to take part) and asked to consent/consent electronically via ticking a box at the bottom of the page. Those that do not tick the box will not be able to access the survey.  **School staff and school Link Worker surveys (WP1, question i (usual provision survey) and WP3, question iii (piloting YES versus signposting)**  After participants have received the information sheet, they will be given a chance to think about the study and to ask questions to the researchers before making a decision about taking part. Consent will be obtained for those that want to participate. This may be via paper or online (via REDCAP).  **Qualitative data (WP3, question iii (piloting YES versus signposting)**  **Pupils**  Young people who were allocated to receive YES or signposting will be contacted by a school staff member not involved in the intervention (e.g. their form tutor) and asked if they are interested in participating an in interview on their experiences. For those that say yes, schools will send out information sheets and consent forms to their parents/guardians (by email) which can be sent back directly to the research team or via the school. There will also be the option for parents/guardians and pupils to complete consent online (via REDCaP), in which case, Information Sheets and Consent forms will be sent via a hyperlink from the research team to the school and then to the parent/guardian. Both the parents/guardians and young people will be given a chance to think about the study and to ask the researchers questions before making a decision about them (or their young person) taking part. If both parent/guardian and young person consent/consent to participate, the researcher will arrange a time to meet with the young person over teams, in person, or phone for the interview.  If it is not possible to obtain a signed consent form from parents/guardians then verbal consent will be taken over the telephone. The researcher will provide a verbal explanation of the study to the parent/guardian (who will already have received the study information sheet), give the parent/guardian the opportunity to ask any questions about the study, and then will read the consent form verbatim to the parent/guardian over the telephone and ask the parent/guardian to verbally agree with each clause, and then state their name and the date at the end if they are happy to consent for their child to take part in the research. The researcher will audio record this conversation. Verbal consent audio recordings will be held in the UCL Data Safe Haven.  **School staff and Link Workers**  For the interviews with school staff or link workers, researchers will provide them with an information sheet. This could be in person or electronically (via email with an attachment or hyperlinkto REDCAP) Participants will be given a chance to think about the study and to ask questions to the researchers before making a decision about taking part. Consent (via a signed email attachment or electronically via REDCAP) to take part from the participants will be taken by the research team. If it is not possible to obtain a signed consent form from school staff or link workers (as may be the case with telephone interviews) then verbal consent will be taken over the telephone, following the procedure outlined above. Verbal consent audio recordings will be held in the UCL Data Safe Haven.  For all data collection, participants will have 1 month to withdraw their data after they have taken part. This is because it will be combined with other data and analysed. This is outlined in all information sheets.  *A copy of your participant information sheet(s) and consent form(s) must be attached to this application. For your convenience proformas are provided in Appendix I. These should be filled in and modified as necessary.*  In cases where it is not proposed to obtain the participants informed consent, please explain why below.  N/A |
| --- | --- | --- |

| \| **C8** \| \| --- \| | **Will any form of deception be used that raises ethical issues? If so, please explain.**  No forms of deception will be used. |
| --- | --- | --- |

| \| **C9** \| \| --- \| | **Will you provide a full debriefing at the end of the data collection phase?**  **Yes**  **No**  If ‘No’, please explain why below.  We will provide school-level feedback on the research at the end of the study to schools. We will not debrief all pupils directly, however, as outlined in the information sheets, results will be posted on the research unit website <https://sbbresearch.org/news-events/> |
| --- | --- | --- |

| \| **C10** \| \| --- \| | **Information Sheets And Consent Forms: Appendix I**  **A poorly written Information Sheet(s) and Consent Form(s) that lack clarity and simplicity frequently delay ethics approval of research projects.** The wording and content of the Information Sheet and Consent Form must be appropriate to the age and educational level of the research participants and clearly state in simple non-technical language what the participant is agreeing to. Use the active voice e.g. “we will book” rather than “bookings will be made”. Refer to participants as “you” and yourself as “I” or “we”. An appropriate translation of the Forms should be provided where the first language of the participants is not English. If you have different participant groups you should provide Information Sheets and Consent Forms as appropriate (e.g. one for children and one for parents/guardians) using the templates provided in Appendix I. Where children are of a reading age, a written Information Sheet should be provided. When participants cannot read or the use of forms would be inappropriate, a description of the verbal information to be provided should be given. Where possible please ensure that you trial the forms on an age-appropriate person before you submit your application. |
| --- | --- | --- |

**nd the researched**

| **SECTION D: DATA STORAGE AND SECURITY** |
| --- |

**SECTIO**

| \| **D1** \| \| --- \| | **Will the research involve the collection and/or use of personal data?**  **Yes**  **No**  **If yes, is the research collecting or using:**   - **sensitive personal data as defined by the UK Data Protection Act (racial or ethnic origin / political opinions / religious beliefs / trade union membership / physical or mental health / sexual life / commission of offences or alleged offences), and/or** - **data which might be considered sensitive in some countries, cultures or contexts?**   **If yes, state whether explicit consent will be sought for its use and what data management measures are in place to adequately manage and protect the data.**  Data collected draws on concepts including community connection, loneliness, mental health and wellbeing. Consent/consent will be sought from all individuals from which data are collected.  Only the INACT research team at University College London will have access to identifiable data.  **Survey data**  **Pupils (WP2, question ii (prevalence rates and predictors) and WP3, question iii (piloting YES versus signposting)**  We will follow the following procedure in order to protect the anonymity of participating pupils: the schools themselves will provide University College London with pupil lists for identified schools. This will include surname, forename, gender, and Unique Pupil Number (UPN). The UPN is unique to each pupil in the NPD. This will be used to create a password for each pupil so that they are able to access the secure online survey. Pupil data will be transferred securely using encryption and stored in the UCL Data Safe Haven which is certified to the ISO27001 information security standard and conforms to NHS Digital's Information Governance Toolkit. The questionnaires completed by pupils are completed within ReDCAP (stored within the UCL Data Safe Haven) and access by a unique link, created by the research team for pupils. The Data Safe Haven can only be accessed by approved members of the INACT team via a dual authentication factor (username and password + phone confirmation).  **School Staff and Link Workers (WP1, question i (usual provision survey) and WP3, question iii (piloting YES versus signposting)**  School staff and Link Workers who complete usual provision and/or implementation surveys will do so via RedCap stored within the UCL Data Safe Haven) and access by a unique link, created by the research team. The Data Safe Haven can only be accessed by approved members of the INACT team via a dual authentication factor (username and password + phone confirmation). They will each be given a unique password to allow them to access the survey.  **Qualitative data (All participants: WP3, question iii (piloting YES versus signposting)**  Young people, Link Workers and school staff who take part in interviews as part of this evaluation will be given a unique ID number that will be used to label their audio files and deidentified transcripts. Participant ID numbers, names, and contact details will be stored in the UCL Data Safe Haven which only the INACT research team can access. Any paper copies of consent/consent forms with participants’ names will be stored in a locked filing cabinet within the Department for Behavioural Science and Health at UCL. |
| --- | --- | --- |
| \| **D2** \| \| --- \| | **During the Project (including the write up and dissemination period)**  **State what types of data will be generated from this project** (i.e. transcripts, videos, photos, audio tapes, field notes, etc).  Survey data  Audio data, de-identified transcripts (qualitative data)  **How will data be stored, including where and for how long?** This includes all hard copy and electronic data on laptops, share drives, usb/mobile devices.  **Survey data**  All survey data will be administered electronically. The questionnaires will not ask for any identifiable data. Electronic consent and consent forms will be stored securely within UCL’s Data Safe Haven. Identifiable data needed for the project to generate survey IDs (such as the schools pupil list and associated unique reference numbers) will be held in separate folders within UCL’s Data Safe Haven.  **Qualitative data:**  With the participant’s permission, interviews will be audio recorded using an encrypted Dictaphone or on Microsoft Teams. Recordings will be uploaded electronically to UCL’s Data Safe Haven; audio recordings will be transcribed verbatim and any identifiable information removed (i.e. names, places); de-identified transcriptions will be held in the UCL Data Safe Haven and audio recordings deleted after quality checks (approx. 1 month after the interview has taken place).  Any paper copies of consent/consent forms will be stored in a locked filing cabinet within the Behavioural Science and Health department at UCL.  **Who will have access to the data, including advisory groups and during transcription?**  Only the INACT research team will have access to the data. |

| \| **D3** \| \| --- \| | **Will personal data be processed or be sent outside of the European Economic Area (EEA)*? No**  **If yes,** please confirm that there are adequate levels of protection in compliance with the DPA 1998 and state what arrangements are below.  ***Please note** that if you store your research data containing identifiable data on UCL systems or equipment (including by using your UCL email account to transfer data), or otherwise carry out work on your research in the UK, the processing will take place within the EEA and will be captured by Data Protection legislation. |
| --- | --- | --- |

| \| **D4** \| \| --- \| | **After the Project**  **What data will be stored and how will you keep it secure?**  Deidentified electronic transcripts and anonymised electronic questionnaire data will be kept for 10 years after the study finishes (October 2035). These will be held on secure servers (S Drive) on UCL.  **Where will the data be stored and who will have access?**  Anonymised electronic data will be held on a secure drive (S Drive) at UCL. Only INACT researchers will have access to data. Requests to access anonymised data only by researchers in the EEA may be requested to a convened approval group consisting of the Co-Principal Investigators (DH and DF) and the Quantitative Senior Research Fellow (FB).  **Will the data be securely deleted?**  **If yes,** please state when will this occur:  Data will be securely deleted 10 years after the study finishes (2035). |
| --- | --- | --- |

| \| **D5** \| \| --- \| | **Will the data be archived for use by other researchers?  Yes  No**  If **Yes**, please describe provide further details including whether researchers outside the EEA will be given access.  Anonymised data can be requested by other researchers after the study has finished. This will go to a convened approval group consisting of the Co-Principal Investigators (DH and DF) and the Quantitative Senior Research Fellow (FB). Researchers outside the EEA will not be given access. |
| --- | --- | --- |

| **SECTION E: DETAILS OF RISKS AND BENEFITS to the researcher and the researched** |
| --- |

| \| **E1** \| \| --- \| | **Please state briefly any precautions being taken to protect the health and safety of researchers and others associated with the project (as distinct from the research participants).**  Researchers will receive qualitative training as part of the study on how to conduct interviews in a calm and collected manner from either Dr Alex Burton (Qualitative researcher) or Dr Dan Hayes (Principal Investigator). If, the researcher finds the interview distressing (for whatever reason), they will be able to contact Dr‘s Hayes or Burton to speak about this further.  A risk assessment has been completed |
| --- | --- | --- |

| \| **E2** \| \| --- \| | **Will these participants participate in any activities that may be potentially stressful or harmful in connection with this research?  Yes  No**  If **Yes**, please describe the nature of the risk or stress and how you will minimise and monitor it.  It is not thought that questions or activities will elicit stress or harm to participants (and have been designed not to invoke these). In the unlikely event that they do, participants will be directed to where they can seek further support (e.g. from school staff and the Samaritans). |
| --- | --- | --- |

| \| **E3** \| \| --- \| | **Will group or individual interviews/questionnaires raise any topics or issues that might be sensitive, embarrassing or upsetting for participants?**  If **Yes,** please explain how you will deal with this.  As mentioned above, it is not thought that questions will elicit stress or harm to participants (and have been designed not to invoke these). In the unlikely event that they do, participants will be directed to where they can seek further support (e.g. from school staff and the Samaritans) |
| --- | --- | --- |
| \| **E4** \| \| --- \| | **Please describe any expected benefits to the participant.**  Participants may benefit from knowing they have provided input into research which aims to understand low community connection and loneliness in young people. Additionally, some people find taking part in research an interesting experience. |

| \| **E5** \| \| --- \| | **Specify whether the following procedures are involved:**  **Any invasive procedure(s)**  **Yes**  **No**  **Physical contact**  **Yes**  **No**  **Any procedure(s) that may cause mental distress**  **Yes**  **No**    Please state briefly any precautions being taken to protect the health and safety of the research participants.  Talking or answering questions about loneliness, mental health, and wellbeing could potentially elicit stress or harm to participants (though this is thought unlikely, given findings from the studies such as Education For Wellbeing ref 6735/009 and 6735/014). In the unlikely event that they do, participants will be directed to where they can seek further support (e.g. School Staff and the Samaritans) |
| --- | --- | --- |

| \| **E6** \| \| --- \| | **Does the research involve the use of drugs?**  **Yes**  **No**  If **Yes**, please name the drug/product and its intended use in the research and then complete Appendix II    **Does the project involve the use of genetically modified materials?**  **Yes**  **No**  If **Yes**, has approval from the Genetic Modification Safety Committee been obtained for work?  Yes  No  If **Yes**, please quote the Genetic Modification Reference Number: |
| --- | --- | --- |

| \| **E7** \| \| --- \| | **Will any non-ionising radiation be used on the research participant(s)?**  **Yes**  **No**  If **Yes**, please complete Appendix III. |
| --- | --- | --- |

| \| **E8** \| \| --- \| | **Are you using a medical device in the UK that is CE-marked and is being used within its product indication?** **Yes**  **No**  If **Yes**, please complete Appendix IV. | |
| --- | --- | --- | --- |
| **CHECKLIST** | |  |

| **Documents to be Attached to Application Form (if applicable) Tick if attached** |
| --- |
| **Section B: Details of the Project**   - Questionnaire(s) / Psychological Tests - Relevant correspondence relating to involvement of collaborating  department/s and agreed participation in the research i.e. approval letters   to gatekeepers seeking permission to do research on their premises/  in their company etc. |
| **Section C: Details of Participants**   - - Parental/guardian consent form for research involving participants under 18   - Participant/s information sheet   - Participant/s consent form/s   - Advertisement |
| **Appendix I: Information Sheet(s) and Consent Form(s)** |
| **Appendix II: Research Involving the Use of Drugs**   - Relevant correspondence relating to agreed arrangements for dispensing   with the pharmacy   - Written confirmation from the manufacturer that the drug/substance has   has been manufactured to GMP     - Proposed volunteer contract - Full declaration of financial or direct interest - Copies of certificates: CTA etc…     **Appendix III: Use of Non-Ionising Radiation**  **Appendix IV: Use of Medical Devices** |

Updated 17.10.2017
